# Salivary aquaporin-3 as a screening biomarker for xerostomia in patients with periodontal disease and the effects of xerostomia on oral health-related quality of life

**DOI:** 10.1371/journal.pone.0283995

**Published:** 2023-04-07

**Authors:** Saira Atif, Norsila Abdul Wahab, Sarah Ghafoor, Ahmad Azlina, Ambreen Tauseef, Sadia Rana, Muhammad Qasim Saeed

**Affiliations:** 1 School of Dental Sciences, Universiti Sains Malaysia Health Campus, Kota Bharu, Kelantan, Malaysia; 2 Combined Military Hospital Lahore Medical College & Institute of Dentistry, National University of Medical Sciences, Lahore, Pakistan; 3 Department of Oral Biology, University of Health Sciences, Lahore, Pakistan; 4 Department of Oral Biology, Sharif Medical and Dental College, Lahore, Pakistan; University of Catania: Universita degli Studi di Catania, ITALY

## Abstract

Xerostomia is a subjective condition of dryness of the oral cavity that may lead to several oral problems deteriorating oral health-related quality of life. This study aimed to (1) determine the prevalence of xerostomia, (2) compare the general health status, unstimulated salivary flow rate, and oral health-related quality of life in xerostomics and non-xerostomics, and (3) investigate the potential of salivary aquaporin-3 (AQP-3) as a screening biomarker for xerostomia in patients with periodontal disease. Demographics and systemic health data were collected from 109 healthy participants, 20 to 55 years old, with Community Periodontal Index (CPI) score ≥ 3. For subjective assessment of xerostomia, Shortened Xerostomia Inventory (SXI) was used. For objective assessment of xerostomia, unstimulated salivary flow rate was measured. Shortened Oral Health Impact Profile (S-OHIP) was utilized for oral health-related quality of life assessment. The collected saliva samples were processed and stored at −80°C. Quantification of salivary AQP-3 protein was done with enzyme-linked immunosorbent assay. Xerostomia was reported in 78% of the subjects based on SXI score. Median concentration of AQP-3 was significantly higher in xerostomics compared to non-xerostomics, p = 0.001. Moreover, oral health-related quality of life was significantly poor in xerostomics compared to non-xerostomics, p = 0.002. Furthermore, there were significant correlations between AQP-3 and SXI (r = 0.21, p = 0.025), AQP-3 and S-OHIP (r = 0.2, p = 0.042), S-OHIP and SXI (r = 0.37, p < 0.001), unstimulated salivary flow rate and random blood glucose level (r = 0.32, p = 0.001), and body mass index and mean arterial pressure (r = 0.44, p < 0.001). Regression analysis showed that body mass index, CPI score 3, and salivary AQP-3 were suitable predictors for presence of xerostomia. AQP-3 could be a potential screening biomarker for xerostomia in patients with periodontal disease for its early identification may help improve oral health-related quality of life of the individuals.

## Background

Xerostomia or dry mouth is a complex phenomenon, defined as a subjective feeling of dryness in the mouth. Hyposalivation may or may not be associated with this oral dryness [1–3]. Xerostomia can occur due to aging, side effects of medications, diabetes mellitus, autoimmune diseases such as Sjögren’s syndrome, psychological disorders, and radiotherapy [4,5]. The global prevalence of xerostomia is 23%, with substantial variation in prevalence reported in different studies [6]. Prolonged xerostomia may increase the incidence of periodontal disease and other oral and dental problems [7], which negatively impact oral health-related quality of life (OHRQOL) [8,9]. In USA, 47% of adults aged 30 years and older suffer from periodontal disease, including gingivitis and periodontitis [10], which is also one of the most common conditions affecting the oral cavity in Southeast Asia [11]. Xerostomia may hinder the effective removal of food particles and microbiological byproducts from the oral cavity and had been indirectly associated with plaque buildup and periodontal disease in young healthy adults [12]. Moreover, the development of periodontitis in people with Sjögren’s syndrome, an autoimmune disease presenting with oral dryness, has been linked to variations in periodontal microcirculation and vascular reactivity [13]. Chronic mouth-breathers experience xerostomia as well, which over time puts them at risk for periodontal disease [14,15]. Additionally, mouth breathers who used supplementary salivary replacements experienced a significant reduction in periodontal inflammation following scaling and root planing [16]. Studies have reported that reduced expression of transglutaminase 1 and 3 proteins in gingival tissue may affect the barrier potential of gingiva to bacterial invasion that is a hallmark of initiation of periodontal disease [17]. Several microRNAs have been recently studied in gingival crevicular fluid of patients with periodontitis and healthy controls and show promising future of these biomarkers in understanding disease progression [18]. Certain proteins have been linked to probing pocket depth in individuals with periodontal disease and xerostomia symptoms [19], which signifies the possibility of discovering a robust screening biomarker for xerostomia in the general population who are at risk of developing periodontal disease and other oral complications of oral dryness.

Saliva is a lubricating biofluid secreted by three pairs of major and numerous minor salivary glands and coats the enamel of the teeth and oral mucous membrane. It performs numerous functions, including food digestion, a barrier against oral microorganisms, buffering potential, prevention of dental caries, wound healing, facilitating speech, and perceiving smell and taste sensations [20]. It is pertinent for oral health owing to its unique composition. The potential utility of whole saliva as a diagnostic medium for oral diseases has been intensively explored over the last few decades as it can be acquired conveniently from patients [21]. In addition to salivary gland secretions, whole saliva also comprises gingival crevicular fluid, serum components, inflammatory cells, desquamated epithelial cells, bronchial and nasal secretions, bacteria and their byproducts, and food particles [22]. Saliva is mostly water, with a small amount of electrolytes, proteins, and other organic substances such as lipids, glucose, urea, and uric acid [23,24]. Protein biomarkers are abundant in saliva and can serve as indicators of a patient’s physiological status. Several salivary biomarkers have been investigated for the diagnosis and treatment outcome of periodontal disease [25,26]. However, there are limited published studies investigating aquaporin-3 (AQP-3) protein as a potential screening biomarker of xerostomia in patients with periodontal disease.

Saliva sampling is done by collecting glandular, stimulated, or unstimulated saliva. The flow rate of saliva that has been stimulated ranges from 1.6–2 ml/min. Flow rate of unstimulated saliva ranges from 0.29 to 0.41 ml/min, while rates below 0.1 ml/min signify hypofunction of salivary glands [27]. In xerostomic patients, the cut-off values for salivary flow rate vary; some authors regard an unstimulated salivary flow rate of greater than 0.25 ml/min to be normal and a value less than 0.25 ml/min indicative of objective xerostomia [28]. Studies have also demonstrated a stronger association of xerostomia with unstimulated salivary flow rate than with stimulated salivary flow rate [29,30]. Severe subjective xerostomia symptoms were also related to a decreased unstimulated salivary flow rate [31]. Unstimulated saliva represents the basal flow rate which covers and shields the oral tissues for almost 14 hours a day [27]. Hence, it is considered most crucial for preserving oral and dental health [32]. In addition to affecting salivary flow rate, xerostomia also led to alterations in the composition of saliva [33,34].

The majority of internal organs including salivary glands use AQPs channels, which are water-permeable transmembrane proteins, to transport solutes and water across cells [35,36]. AQPs are crucial in the physiological and pathological responses of salivary glands. Human salivary gland cells express several AQPs including AQP-1, AQP-3, AQP-4, AQP-5, AQP-6, and AQP-7 [37]. Alterations in AQP expression have been linked to diseases and conditions such as Sjögren’s syndrome, diabetes mellitus, salivary gland tumors, recent exposure to radiation therapy, and physiological aging that elicit xerostomia symptoms and hypofunction of the salivary glands [38]. Recently, initiatives were taken to explore possible biomarkers in saliva linked to xerostomia in particular illnesses, treatments, or old age. AQP-3 had been proposed to play an essential role in the secretion of saliva [39]. In minor salivary glands, AQP-3 staining intensity and localization were reported to be different among these three groups: Patients with Sjögren’s syndrome, patients with subjective xerostomia, and healthy controls [39]. Additionally, it has been suggested that AQP-3 and AQP-5 expression as well as the degree of immunoreactivity in the labial salivary glands could be used to differentiate between people who are systemically healthy from those who have objective and subjective xerostomia, or Sjögren’s syndrome. Most studies on AQP-3 in humans were performed using biopsy specimens of minor salivary glands for the diagnosis of Sjögren’s syndrome. Saliva is an appropriate diagnostic medium for discovering and researching AQP-3 in healthy persons without any underlying medical conditions. Additionally, a comprehensive picture of its function in oral disease screening may be known if AQP-3 is quantified in saliva representing its secretion in the distinctive oral cavity milieu. Saliva sampling is also preferred in situations requiring extensive screening since it eliminates the need for blood collection, serum separation, and sample processing and storage; hence, it saves time, effort, and cost.

The current method of xerostomia screening is subjective based on validated questionnaires. However, using this method, xerostomia may be reported without reduced salivary flow rate, making these screening methods questionable. Discovering potential biomarkers for xerostomia is highly warranted to improve quality of life. There are limited studies on subjective xerostomia prevalence, salivary flow rates, salivary biomarkers of xerostomia, and its effect on OHRQOL in systemically health adults. Moreover, Pakistan is a developing country with limited resources, shortage of trained dentists, and unavailability of cost-effective dental checkup facilities for the general population. In such situations, saliva is a suitable fluid for mass screening of xerostomia for its early identification and rectification. Identification of salivary biomarkers such as AQP-3 as a screening tool may turn out to be a significant move towards developing point-of-care testing for xerostomia in the future. This study aimed to determine the prevalence of xerostomia in patients with periodontal disease and to compare the general health status, unstimulated salivary flow rates, and OHRQOL in xerostomics and non-xerostomics. This study also intended to investigate the potential of using salivary AQP-3 as a screening biomarker for xerostomia.

## Materials and methods

### Study design and sample

This cross-sectional study was conducted on systemically healthy adult participants with periodontal disease attending the Combined Military Hospital (CMH) Lahore Medical College & Institute of Dentistry, Lahore, Pakistan from February 2018 to February 2019. Enzyme-linked immunosorbent assay (ELISA) was performed at Oral Biology Laboratory, University of Health Sciences, Lahore, Pakistan. Ethical approvals were obtained before the start of the study from the Ethical Review Committees of Universiti Sains Malaysia and CMH Lahore Medical College & Institute of Dentistry (reference numbers: USM/JEPeM/18070293 and 74/ERC/CMH/LMC, respectively) according to the Declaration of Helsinki.

Through systematic random sampling, 109 participants were included. The inclusion criteria were as follows: (i) age ≥ 20 years, (ii) Community Periodontal Index (CPI) score ≥ 3 and (iii) at least 4 out of 6 sextants suitable for probing as per CPI criteria. CPI method was used for periodontal health status evaluation as it addresses the hierarchical aspect of periodontal disease and is commonly used for periodontal screening and to measure community oral health [40]. The exclusion criteria were as follows: (i) overall CPI score ≤ 2, (ii) systemic or autoimmune disease, (iii) oral mucosal inflammatory condition, (iv) smoking or betel nut chewing habits, (v) medications, (vi) pregnancy or lactating mothers, and (vii) history of scaling and root planing in the last 12 months. Informed written consent was obtained from all participants and collected data were kept confidential.

### Sociodemographic characteristics and general health status

Sociodemographic characteristics (age, sex, monthly household income, educational attainment) and general health status [body mass index (BMI), mean arterial pressure (MAP), random blood glucose level] were recorded. Blood pressure was measured with Omron digital blood pressure monitor (M10-IT, Omron Healthcare, Kyoto, Japan) and MAP was calculated by adding systolic blood pressure and two times diastolic blood pressure, then divided by three. Random blood glucose level was measured with Accuchek® Performa digital glucometer (Roche Diagnostics, Mannheim, Germany). Blood pressure and random blood glucose level were taken twice by an outpatient department nurse on the day of data collection and the average values were recorded.

### Assessment of xerostomia

For xerostomia evaluation, Shortened Xerostomia Inventory (SXI) was utilized [41]. Participants were asked to score their recent experience of dry mouth through five questions on a Likert scale of 1 (never) to 5 (always) using a three-response format [42,43] with a total SXI score ranging from 5 to 15. Each study participant was labeled as xerostomic or non-xerostomic based on SXI score; SXI > 5 as xerostomic and SXI = 5 as non-xerostomic.

### Assessment of OHRQOL

Shortened Oral Health Impact Profile (S-OHIP) was utilized [44] to assess the impact of oral health problems on the participant’s quality of life during the previous 12 months. The questionnaire consisted of 14 questions with a five-point Likert scale response format with a score ranging from zero to 56.

### Collection of unstimulated saliva

After administration of SXI, whole unstimulated saliva was collected. Saliva was collected, processed, and stored using the procedures indicated by Henson and Wong [45]. Before saliva collection, each participant was requested to fast for at least an hour, refrain from drinking anything, or conduct any oral hygiene tasks. Saliva was collected in a sterile conical centrifuge tube (SPL Life Sciences, Korea) between 8 and 10 in the morning. The tube and funnel were weighed before collecting saliva. The participant received a complete explanation of the saliva collection process. Participant was given distilled water to thoroughly rinse the mouth for one minute. After five minutes, the participant was instructed to sit straight, incline his or her head slightly forward, and refrain from moving or talking while collecting saliva. The participant received a collection tube with a funnel in a 100 ml cup containing crushed ice. In the beginning, the participant was asked to swallow to clear the mouth of saliva. The participant was further instructed to passively drool saliva into the tube’s funnel without swallowing the saliva for five minutes and to spit any residual saliva into the tube at the end of five minutes. The saliva collection tube and funnel were weighed again. The weight of the saliva was recorded and unstimulated salivary flow rate was calculated in ml/min, considering that 1 g = 1 ml of saliva [3,45]. The collected saliva samples were immediately centrifuged for 15 minutes at 2500 x g at 4°C (Z 400 K, Hermle Labor-Technik, Germany) [46] and supernatants were stored in pre-chilled cryovials (SPL Life Sciences, Korea) at −80°C (Biobase, China) until further analysis using ELISA method.

### Evaluation of periodontal health status

After saliva collection, CPI scores were recorded with WHO periodontal probe (CPI score 3: presence of periodontal pocket of 3.5–5.5 mm; CPI score 4: presence of periodontal pocket of ≥ 6 mm [40]. The participant’s overall CPI score was determined by the highest CPI score of the sextants. A single researcher performed all CPI scorings of all study participants. Intraclass correlation coefficient (ICC) was 0.96 for intra-examiner reliability of CPI scoring.

### Quantification of AQP-3 using ELISA

Supernatant saliva samples were thawed to room temperature before use and briefly vortexed. To quantify AQP-3 protein, 96-well micro-ELISA plates were used according to the manufacturer’s protocol (Elabscience Biotechnology Inc., USA). The detection range of the AQP-3 ELISA kit was 0.31–20 ng/ml. All saliva samples were run in duplicate.

### Data analyses

The data were analyzed using IBM SPSS software version 27. Data of 109 participants were analyzed. Normality of the data was assessed using Shapiro-Wilk’s test. Non-parametric tests were utilized for analyses. Continuous variables were presented as median and interquartile range (IQR). Categorical variables were described as frequency and percentage. Results were compared between groups using Mann-Whitney U test, Chi-squared test, or Fisher’s exact test. The correlations between variables were analyzed using Spearman matrix correlation. A binary logistic regression model was generated with the presence of xerostomia as predictor variable and age, sex, BMI, MAP, random blood glucose level, S-OHIP score, unstimulated salivary flow rate, CPI, and salivary AQP-3 concentration as explanatory variables. P value < 0.05 was considered significant.

## Results

The study comprised 109 patients with periodontal disease, who were 20–55 years old. Sociodemographic characteristics, general health status, periodontal status, unstimulated salivary flow rate, and S-OHIP scores are presented in Table 1. Median age of the study participants was 27 years. About 54% of the study participants had moderate socioeconomic status (SES), including 19.3% of males and 34.9% of females. About 24% of the study participants had received high school diploma (Grade 10). Males had a higher MAP compared to females (p = 0.009). Moreover, females had significantly poor OHRQOL compared to males (p < 0.001).

**Table 1:** Sociodemographic, general health, periodontal status, unstimulated salivary flow rate, and S-OHIP scores of patients with periodontal disease.

Variable	n	Male	n	Female	p value	n	All
**Median age (IQR) in years**	47	29 (24–39)	62	25.5 (20–36)	0.086^⸙^	109	27 (21.5–36)
**% Very low SES** †	1	0.9%	4	3.7%	0.152^**#**^	5	4.6%
**% Low SES**	18	16.5%	14	12.8%		32	29.4%
**% Moderate SES**	21	19.3%	38	34.9%		59	54.1%
**% High SES**	7	6.4%	6	5.5%		13	11.9%
**% Education < 5 years** ^§^	2	1.8%	13	11.9%	**0.001** * ^**#**^	15	13.8%
**% Grade 5**	-	-	3	2.8%		3	2.8%
**% Grade 8**	13	11.9%	10	9.2%		23	21.1%
**% Grade 10**	12	11%	14	12.8%		26	23.9%
**% Grade 12**	10	9.2%	20	18.3%		30	27.5%
**% Undergraduate degree**	9	8.3%	1	0.9%		10	9.2%
**% Postgraduate degree**	1	0.9%	1	0.9%		2	1.8%
**Median BMI (IQR)**	47	22.4 (21–25.6)	62	22.8 (20.4–27.4)	0.586^⸙^	109	22.6 (20.8–26.4)
**Median MAP (IQR) in mmHg**	47	95.3 (90–102)	62	90.8 (86.7–98.8)	**0.009** * ^⸙^	109	92 (87.7–100)
**Median random blood glucose level (IQR) in mg/dl**	47	104 (95–115)	62	102.5 (98–111.3)	0.755^⸙^	109	103 (98–112.5)
**Median S-OHIP score (IQR)**	47	11 (7–14)	62	20 (12–26.3)	**<0.001** * ^⸙^	109	15 (9.5–22.5)
**Median unstimulated salivary flow rate (IQR)**	47	0.42 (0.27–0.6)	62	0.43 (0.32–0.57)	0.854^⸙^	109	0.43 (0.29–0.59)
**% CPI 3**	30	27.5%	47	43.1%	0.174^^^	77	70.6%
**% CPI 4**	17	15.6%	15	13.8%		32	29.4%

BMI, body mass index; CPI, Community Periodontal Index; IQR, interquartile range; MAP, mean arterial pressure; SES, socioeconomic status; S-OHIP, Shortened Oral Health Impact Profile.

†Socioeconomic status used based on the study by Warsi *et al*. [47].

^§^Educational attainment based on the education system of Pakistan.

^^^Results from Chi-squared test.

^#^Results from Fisher’s exact test.

^⸙^Results from Mann-Whitney U test.

*Significant at p = 0.05 level.

According to the SXI score, 85 participants (78%) reported having xerostomia, while 24 participants (22%) did not. Table 2 provides the participants’ frequency distribution and overall SXI score.

**Table 2:** Overall SXI score and participant frequency distribution.

SXI score	Frequency (n)	%
**5**	24	22
**6**	29	26.6
**7**	24	22
**8**	12	11
**9**	7	6.4
**10**	8	7.3
**11**	4	3.7
**12**	1	0.9

Table 3 presents the results of associations using Mann-Whitney U or Chi-squared tests, between xerostomia status and age, sex, general health status, periodontal status, unstimulated salivary flow rate, and salivary AQP-3 concentration. BMI was significantly lower in the xerostomic group compared to non-xerostomic group, p = 0.015. Moreover, S-OHIP scores were significantly higher in the xerostomic group compared to non-xerostomic group, p = 0.002 indicating that OHQROL was poor in the xerostomics compared to non-xerostomics. There was also a significant association between CPI and xerostomia status, p = 0.012, and majority of the xerostomics had CPI score 3. However, unstimulated salivary flow rate was not significantly reduced in the xerostomics compared to non-xerostomics, p = 0.46. In xerostomics, the median concentration of AQP-3 was 0.53 (0.4–0.89) ng/ml and in non-xerostomics was 0.39 (0.35–0.51) ng/ml. According to Mann-Whitney U test, salivary AQP-3 protein concentration was significantly higher in xerostomic compared to non-xerostomic groups, p = 0.001.

**Table 3:** Association of xerostomia status with age, sex, general health status, OHRQOL, unstimulated salivary flow rate, periodontal status, and salivary AQP-3 concentration in patients with periodontal disease.

Variable	n	Xerostomia	n	Non-xerostomia	p value	n	All
**Median age (IQR) in years**	85	27 (21–35.5)	24	33 (22.3–40)	0.164^⸙^	109	27 (21.5–36)
**% Female**	52	47.7%	10	9.2%	0.088^	62	56.9%
**% Male**	33	30.3%	14	12.8%		47	43.1%
**Median BMI (IQR)**	85	22.5 (20.3–25.5)	24	24.4 (22–28.9)	**0.015** * ^⸙^	109	22.6 (20.8–26.4)
**Median MAP (IQR) in mmHg**	85	91.3 (87–100)	24	95 (89–101.6)	0.261^⸙^	109	92 (87.7–100)
**Median random blood glucose level (IQR) in mg/dl**	85	102 (97.5–110.5)	24	108.5 (98–127.8)	0.065^⸙^	109	103 (98–112.5)
**Median S-OHIP score (IQR)**	85	17 (11–23.5)	24	9.5 (4.3–16.5)	**0.002** * ^⸙^	109	15 (9.5–22.5)
**Median unstimulated salivary flow rate (IQR)**	85	0.42 (0.29–0.59)	24	0.49 (0.31–0.6)	0.460^⸙^	109	0.43 (0.29–0.59)
**% CPI 3**	65	59.6%	11	9.1%	**0.012** * ^	77	70.6%
**% CPI 4**	20	18.3%	11	9.1%		32	29.4%
**Median AQP-3 (IQR) in ng/ml**	85	0.53 (0.4–0.89)	24	0.39 (0.35–0.51)	**0.001** * ^⸙^	109	0.48 (0.38–0.81)

AQP, aquaporin; BMI, body mass index; CPI, Community Periodontal Index; IQR, interquartile range; MAP, mean arterial pressure; S-OHIP, Shortened Oral Health Impact Profile.

^^^Results from Chi-squared test.

^⸙^Results from Mann-Whitney U test.

*Significant at p = 0.05 level.

Table 4 presents Spearman’s matrix correlation analyses between variables. There were significant correlations between AQP-3 and SXI, AQP-3 and S-OHIP, SXI and S-OHIP, MAP and BMI, and random blood glucose level and unstimulated salivary flow rate.

**Table 4:** Correlations between variables in patients with periodontal disease.

Variable	AQP-3	MAP	RBG	BMI	S-OHIP	CPI	USFR
**MAP**	0.01, 0.89						
**RBG**	−0.06, 0.553	0.18, 0.065					
**BMI**	−0.15, 0.128	**0.44, <0.001** **	0.17, 0.073				
**S-OHIP**	**0.2, 0.042** *	−0.18, 0.064	−0.05, 0.625	−0.14, 0.151			
**CPI**	−0.02, 0.812	0.16, 0.096	0.04, 0.718	0.05, 0.601	−0.06, 0.536		
**USFR**	−0.09, 0.332	−0.12, 0.2	**0.32, 0.001** **	0.01, 0.958	0.05, 0.585	0.03, 0.746	
**SXI**	**0.21, 0.025** *	−0.15, 0.116	−0.07, 0.48	−0.18, 0.064	**0.37, <0.001** **	−0.16, 0.104	0.07, 0.462

AQP, aquaporin; BMI, body mass index; CPI, Community Periodontal Index (score 3 and 4); MAP, mean arterial pressure; RBG, random blood glucose level; S-OHIP, Shortened Oral Health Impact Profile; SXI, Shortened Xerostomia Inventory; USFR, unstimulated salivary flow rate.

Spearman matrix correlation test used, results presented as correlation coefficient (r) and p value.

**Correlation is significant at p = 0.01 level (2-tailed).

*Correlation is significant at p = 0.05 level (2-tailed).

Table 5 presents results of the binary regression model. Initially, all variables were added to the model, and the final model excluded all variables presenting alpha ≥ 10. By doing so, the three explanatory variables remaining in the model were BMI, CPI score 3, and AQP-3.

**Table 5:** Binary logistic regression analysis of presence of xerostomia in patients with periodontal disease.

Presence of xerostomia	Initial model			Final model		
	Coefficient	p value	OR (95% CI)	Coefficient	p value	OR (95% CI)
**Female**	0.43	0.524	1.53 (0.33–3.31)			
**Age**	−0.005	0.89	1 (0.93–1.07)			
**BMI**	−0.15	0.061	0.86 (0.73–1.01)	−0.16	**0.017** *	0.85 (0.75–0.97)
**MAP**	0.02	0.607	1.02 (0.94–1.11)			
**Random blood glucose level**	−0.02	0.311	0.98 (0.94–1.02)			
**S-OHIP**	0.03	0.4	1.03 (0.96–1.11)			
**Unstimulated salivary flow rate**	−0.34	0.792	0.71 (0.06–9.01)			
**CPI 3**	1.25	**0.038** *	3.5 (1.07–11.39)	1.38	**0.012** *	3.96 (1.35–11.6)
**AQP-3**	3.36	**0.024** *	28.81 (1.55–535.39)	3.76	**0.009** *	42.93 (2.58–715.31)
**R** ^**2**^	37.2%			32.6%		

AQP, aquaporin; BMI, body mass index; CI, confidence interval (with upper and lower limit given in parenthesis); CPI, Community Periodontal Index; MAP, mean arterial pressure; OR, odds ratio; R^2^, Nagelkerke R squared; S-OHIP, Shortened Oral Health Impact Profile.

The final model explained 32.6% of the variance in presence of xerostomia and correctly predicted 92.9% of xerostomic cases and 25% of non-xerostomia cases, with overall accuracy of the model as 78%.

*Significant at p = 0.05 level.

## Discussion

In this study, the data of 109 patients with CPI ≥ 3 from Lahore, Pakistan were analyzed. There is little data available on the prevalence of xerostomia in Pakistan. Findings from this study revealed that majority of the study participants were xerostomics. Mizutani *et al*. [12] and Thomson *et al*. [48] have also reported that 70% and 78.8% of their study participants, respectively, were xerostomics depending on their answer to the question related to frequency of experiencing mouth dryness: ‘occasionally’, ’very often’, or ’frequently’. The authors categorized a person as xerostomic only if he or she responded to this question with ’very often’ or ’frequently’, whereas participants responding with ‘occasionally’ experiencing xerostomia were considered as non-xerostomic. Similar to this current study, Thomson et al. [1] also reported a higher prevalence of ‘occasional’ xerostomia among their study participants. Hence, the differences in the reported prevalence of xerostomia in different studies obtained using different assessment measures such as validated questionnaires with or without flow rate assessment were not surprising as there is no universal method of labeling a person as xerostomic. Participants of this study were labeled as xerostomics based on their SXI score of > 5. Various methods of xerostomia assessment have been utilized by researchers besides SXI such as Xerostomia Inventory (XI), Xerostomia Questionnaire (XQ), and response to a single standard xerostomia question [1,12,48–50]. Due to the categorization of xerostomia based on SXI > 5 in this study, variability in perceptions of xerostomia may be different from other study populations. The participants of this study might have a higher prevalence of xerostomia as a result of difference in their lifestyle and dietary habit of increased coffee or tea consumption. Drinking caffeinated beverages is known to have diuretic effects resulting in increased mouth dryness [12].

This study found no significant relationship between xerostomia status and sex, which is consistent with earlier investigations [33,48,51]. Few authors have reported that women are more likely than men to experience xerostomia [50,52,53]. In older populations, often those over 50 years old, a significant relationship between xerostomia status and sex has been reported, with higher prevalence in females [50,54]. In contrast, the participants in this study were young and middle-aged adults. Furthermore, the lack of a relationship between xerostomia and sex in this study could be due to genetic and ethnic factors.

In this study, the association was not found between xerostomia status and age which was similarly reported by others [1,53]. Nevertheless, some authors have reported a significant association [50]. According to Niklander *et al*., the likelihood of xerostomia increased 1.01 times for every one-year increase in age [50]. The different findings could be observed as the participants of this study were not on any medications, in contrast to 42.4% of those in the Niklander *et al*. study who were on medication.

The median S-OHIP scores in patients with periodontal disease were in accordance with others [55–57]. Moreover, the present study corroborates with others that periodontal disease negatively impacts OHRQOL [58,59]. In this study, the association between S-OHIP scores and xerostomia status was significant, indicating poor OHRQOL of the xerostomics. Moreover, there was a significant and positive correlation between SXI and S-OHIP scores which is similarly reported by others [1,28]. Though the correlation was not strong, it may suggest that an increase in the SXI score represented severity of xerostomia which may have resulted in poor OHRQOL.

Majority of the study participants had unstimulated salivary flow rate within the normal reported range. Published studies corroborate the findings of this study that hyposalivation was not observed in patients with periodontal disease. The salivary flow rate of the participants with periodontal disease was comparable to that of the general population [22] and also to patients reporting periodontitis [60]. Unstimulated salivary flow rate in individuals with periodontal disease had been observed to be 0.5 ml/min [61,62]. In another study, patients with stage III periodontitis had a mean salivary flow rate of 0.34±0.16 ml/min [63], which was lower than reported by others; however, this is still within the normal salivary flow rate range and could be due to the stage of periodontal disease. It was also interesting to note that there was a significant and positive correlation between unstimulated salivary flow rate and random blood glucose level among the study participants. There is scarce data available on correlation between these two variables in healthy individuals. Authors have reported a negative correlation between HbA1c level and unstimulated parotid salivary flow rate of their 39 study participants comprising 24 patients with diabetes and 15 non-diabetic controls. The difference could be due to smaller sample size, presence of diabetes, difference in participants age, and type of saliva [64].

Unstimulated salivary flow rate was not different between the xerostomics and non-xerostomics in this study. Similar results were reported by others [50,65–68]. The results of this study also validated that xerostomics may not experience concurrent hyposalivation and objective and subjective xerostomia may not be in accordance. Oral dryness is clinically manifested when the salivary flow rate is reduced by at least 40% [69]. Current understanding distinguishes subjective xerostomia from hyposalivation as xerostomic individuals may report normal or even high salivary flow rates [34]. This study, in line with earlier investigations, found no correlation between unstimulated salivary flow rate and SXI [70]. Others have reported a weak correlation between flow rate and xerostomia [71,72]. The difference in the study design, study population, and underlying systemic diseases could have resulted in varied results.

To the best of our knowledge, the concentration of salivary AQP-3 has not been previously reported in systemically healthy xerostomics with periodontal disease. This study confirmed that salivary glands express and secrete AQP-3 proteins in whole saliva. Moreover, participants with xerostomia had higher salivary levels of AQP-3 than participants who were non-xerostomics. According to Ichiyama *et al*. [39], mRNA expression of AQP-3 was central for saliva secretion and compared to healthy controls, patients with Sjögren’s syndrome and dry mouth had lower mRNA expression but higher immunoreactive intensities of AQP-3 in their salivary glands [39]. In this study, AQP-3 concentration was higher in xerostomics compared to non-xerostomics, which could be because the present study evaluated secreted protein levels in saliva compared to mRNA expression in salivary gland tissue samples in the other study. The secreted protein level may not correspond with mRNA expression due to post-translational and post-transcriptional pathways [73,74]. Furthermore, modest sample size and use of a tissue sample from only the minor salivary glands could also have contributed to the varied results.

The concentration of salivary AQP-3 protein increased with SXI and S-OHIP scores. These correlations had not been previously reported. Even though the correlations were not strong, it could indicate that xerostomia had an impact on the amount of AQP-3 protein released in saliva which could then be quantitatively assessed. Moreover, increase in AQP-3 protein in saliva also indicates poor OHRQOL. Further research on these aspects is required. Binary logistic regression showed that an increase in AQP-3 protein concentration, a decrease in BMI, and periodontal status of CPI score 3 also increased the risk of having xerostomia. It is interesting to note that for every one-unit increase in salivary concentration of AQP-3, the odds of being xerostomic increased 42.93 times and for every one-unit decrease in BMI, the odds of having xerostomia increased 0.85 time. Participants with CPI score 3 and a negative association between BMI and presence of xerostomia presented here are intriguing and deserves future investigations. However, the CI was large for AQP-3 which might be the result of data bias due to fewer non-xerostomic than xerostomic participants.

This study has established that there are changes in the salivary AQP-3 levels in xerostomics compared to non-xerostomics. However, there are certain limitations. The participants of this study were from Lahore, the provincial capital of Punjab in Pakistan. This province has a different climate than the other three provinces of Pakistan. Moreover, compared to people living in other urban areas or rural places of Pakistan, Lahore city dwellers may have different behaviors and perceptions of oral dryness which may have affected the results of prevalence of xerostomia. Hence, participants from other cities and provinces should be included in future studies for representative sampling and more generalizable results. Additionally, as the goal of this study was to exclusively examine xerostomia in systemically healthy adults with periodontal disease, it might have resulted in under-representation of older participants or severe xerostomics because of exclusion criteria. Severity of xerostomia worsens in old age, impairing the quality of life in the elderly [75]. Therefore, it is advised that future research examines xerostomia in individuals from all age ranges. Moreover, CPI method was used in this study for periodontal health status assessment as the index is inherently simple to perform, reproducible, and less time-consuming especially when a single researcher collects all data and where mass screening is required. It is recommended that future researchers also evaluate salivary AQP-3 protein concentrations in patients with chronic periodontitis utilizing the recent classification of periodontitis.

## Conclusion

Patients with periodontal disease had a high prevalence of subjective xerostomia based on SXI, even though the objective measurement using unstimulated salivary flow rate was within the normal range. Salivary AQP-3 protein concentration was higher in participants with xerostomia compared to non-xerostomia, providing valuable insight for its potential as a xerostomia biomarker which should be further explored. Moreover, OHRQOL deteriorated in the xerostomics. Further research is warranted to validate AQP-3 as a screening biomarker for xerostomia in patients with periodontal disease.

## Supporting information

S1 Dataset Study participants original dataset. (XLSX)
